# A *Gaussia* Luciferase Cell-Based System to Assess the Infection of Cell Culture- and Serum-Derived Hepatitis C Virus

**DOI:** 10.1371/journal.pone.0053254

**Published:** 2012-12-31

**Authors:** George Koutsoudakis, Sofía Pérez-del-Pulgar, Patricia González, Gonzalo Crespo, Miquel Navasa, Xavier Forns

**Affiliations:** Liver Unit, Institut D'Investigacions Biomèdics August Pi i Sunyer, Centro de Investigación Biomédica en Red: Enfermedades Hepáticas y Digestivas, Hospital Clínic, Barcelona, Spain; Inserm, U1052, UMR 5286, France

## Abstract

Robust replication of hepatitis C virus (HCV) in cell culture occurs only with the JFH-1 (genotype 2a) recombinant genome. The aim of this study was to develop a system for HCV infection quantification analysis and apply it for the selection of patient sera that may contain cell culture infectious viruses, particularly of the most clinically important genotype 1. Initially, a hepatoma cell line (designated Huh-7.5/EG(4A/4B)GLuc) was generated that stably expressed the enhanced green fluorescent protein (EGFP) fused in-frame to the secreted *Gaussia* luciferase via a recognition sequence of the viral NS3/4A protease. Upon HCV infection, NS3/4A cleaved at its signal and the *Gaussia* was secreted to the culture medium, thus facilitating the infection quantification. The Huh-7.5/EG(4A/4B)GLuc cell line provided a rapid and highly sensitive quantification of HCV infection in cell culture using JFH-1-derived viruses. Furthermore, the Huh-7.5/EG(4A/4B)GLuc cells were also shown to be a suitable host for the discovery of anti-HCV inhibitors by using known compounds that target distinct stages of the HCV life cycle; the Ź-factor of this assay ranged from 0.72 to 0.75. Additionally, eighty-six sera derived from HCV genotype 1b infected liver transplant recipients were screened for their *in vitro* infection and replication potential. Approximately 12% of the sera contained *in vitro* replication-competent viruses, as deduced by the *Gaussia* signal, real time quantitative PCR, immunofluorescence and capsid protein secretion. We conclude that the Huh-7.5/EG(4A/4B)GLuc cell line is an excellent system not only for the screening of *in vitro* replication-competent serum-derived viruses, but also for the subsequent cloning of recombinant isolates. Additionally, it can be utilized for high-throughput screening of antiviral compounds.

## Introduction

Hepatitis C virus (HCV), which infects 2–3% of the world’s population, is a major cause of chronic hepatitis, leading to liver cirrhosis and hepatocellular carcinoma in a significant portion of infected patients [1]. HCV is an enveloped positive-strand RNA virus that belongs to the *Flaviviridae* family [2]. The genome of HCV is composed of the 5′ non-translating region (5′ NTR), a single open reading frame encoding at least 10 proteins and the 3′ NTR. The viral particle is composed of structural proteins, core (C), and the envelope glycoproteins (E1 and E2). The other non-structural proteins (NS proteins) include the viroporin ion channel p7, the NS2–3 protease, the NS3 dual-function protein (serine protease and helicase), the NS4A polypeptide, the NS5A phosphoprotein and the NS5B RNA-dependent RNA polymerase (RdRp) [3]. There are six distinct HCV genotypes and multiple subtypes [4]; among these genotypes there exist clusters of global distribution, with types 1a and 1b being the most common, accounting for about 60% of global infections [5].

*In vitro* HCV studies advanced through two breakthroughs: first, subgenomic replicons of subtypes 1b [6], [7] and 1a [8], which replicate autonomously and preferably in selected subclones of the human hepatoma cell line Huh-7, proved to be highly permissive for HCV replication; e.g., Huh-7.5 [9] or Lunet cells [10]; second, the JFH-1 (genotype 2a) isolate, which supports a full infectious cycle in cell culture [11], as well as in its intra- and inter-genotypic chimeric derivatives (e.g., the JC1 chimera) [12], [13], [14]. Although propagation of HCV in cell culture has been an important contribution to the field, it is generally recognized that while subgenomic replicons do exist for a limited number of strains, only the JFH-1 isolate completes the HCV life cycle *in vitro*. Hence, the development of new *in vitro* replication-competent isolates became a priority.

Methodologies and detection methods for HCV *in vitro* infection have ranged from immunostaining and quantitative PCR to the use of infectious viruses carrying reporter genes (e.g., *Firefly* luciferase or Green Fluorescent Protein [GFP]) [15], [16]. Overall, cell-based assays which depend on viral enzymes appear advantageous to those assays that are based on bulk populations in terms of offering a mean for differentiating between viral and cellular functions. Lee *et al.* [17] developed a cell-based assay for monitoring HCV NS3/4A protease activity in mammalian cells. Their study described a substrate vector in which the enhanced GFP (EGFP) was fused to the secreted alkaline phosphatise (SEAP) through the NS3/4A protease decapeptide recognition sequence, which spans the NS4A and NS4B junction region (Delta4AB). Mammalian cells stably expressing the EGFP-Delta4AB-SEAP cassette enabled the monitoring of NS3/4A activity upon expression *in trans* of the protease by subgenomic HCV replicon transfection. Iro *et al*. [18], created a Huh7 cell line (designated Huh7-J20) expressing the identical cassette for rapid and sensitive quantification of HCV infection in cell culture by JFH-1 or JFH-1 chimeric viruses. Finally, Pan et al *et al*. [19], generated a similar stable cell line (designated Huh7.5-EG(Δ4B5A)SEAP), which is based on the Huh-7.5 cells and the NS4B-NS5A junction region as a recognition sequence for the viral protease .

Recently, a real-time imaging of HCV infection using a fluorescent cell-based reporter system has been described [20]. In this system a cellular marker of HCV infection was constructed based on a known substrate of the NS3/4A protease, the mitochondrially tethered interferon (IFN)-β promoter stimulator protein 1 (IPS-1). Huh-7.5 cells stably express a chimeric protein encompassing the C-terminal region of IPS-1 fused to diverse fluorescence proteins alone (Fluorescence Proteins-IPS) or via a nuclear localization sequence (Fluorescence Proteins-NLS-IPS). Chimeric proteins localization followed a mitochondrial pattern along with the single IPS-1 localization. Upon JFH-1 infection or replication of subgenomic replicons of diverse subtypes, expression of the viral protease lead to the IPS substrate recognition and truncation and subsequent re-localization of the fluorescent proteins in the whole cytoplasm or in the nucleus (for those constructs that contained the NLS). This system allowed the monitoring of HCV infection at a single cell level by live-cell imaging of viral propagation.

The aim of the present study was to develop a robust and highly sensitive system for HCV infection quantification analysis. Given the advantages of the NS3/4A protease systems described above, we established a new cell line, designated Huh-7.5/EG(4A/4B)GLuc, which stably expresses the construct described by Lee *et. al.* modified as follows: EGFP was fused to the robust *Gaussia* luciferase [21] via a recognition sequence for the NS3/4A protease. The utility of this new system was evaluated not only in terms of virus entry and replication inhibition by means of JFH-1 infections and known inhibitors neutralizations, but also for the screening of clinical sera with the capacity to contain in *vitro* replication-competent isolates.

## Materials and Methods

### Ethics Statement

The Investigation and Ethics Committee of Hospital Clinic Barcelona approved our protocol, including the use of human samples, which conformed to the ethical guidelines of the 1975 Declaration of Helsinki. Written informed consent was obtained from all the patients included in this study.

### Cell Culture and Cell Lines

Huh-7.5 [9] (kindly provided by Prof. Charles Rice, The Rockefeller University, NY, USA), and 293T (HEK293T cells, American Type Culture Collection, Manassas, VA, CRL-1573) cells were grown in Dulbecco's modified Eagle medium (DMEM; Invitrogen, Carlsbad, CA) supplemented with 2 mM L-glutamine, non-essential amino acids, 100 U of penicillin per ml, 100 µg of streptomycin per ml, and 10% fetal calf serum (FCS), designated DMEM complete, in an incubator with 5% CO_2_ at 37°C.

### Plasmid Construction and Establishment of the Gaussia Cell Line, Huh-7.5/EG(4A/4B)GLuc

This method is provided in the *Supporting Information S1* section.

### HCVcc Infections and Neutralizations

JC1 virus was prepared as described [22] and used to inoculate Huh-7.5/EG(4A/4B)GLuc cells for 4 h previously seeded in 96 well plates, 1.2×10^4^ cells/well. In the case of IFN-α (Interferon-αA, Sigma-Aldrich, St. Louis, MO), 10 h post-seeding, cells were washed 1x with PBS and fed with fresh DMEM complete supplemented with the indicated doses of IFN-α for 8 h. In the case of anti-receptor inhibitions (anti-CD81 mouse mAb clone JS-81, BD Pharmingen, San Diego, CA; or anti-SR-BI, clone C167, kindly provided by Dr. A. Nicosia), cells were treated with the indicated doses for 1 h prior to infection. Finally, viruses were incubated with the indicated amounts of anti-E2 human conformational mAb AR3A (kindly provided by Dr. M. Law) for 1 h at RT with gentle agitation prior to inoculation. Infections were performed always in duplicate wells measured in duplicates (n = 4). Results are given as percentage relative to control infections as follows: IFN-α relative to mock infections, anti-CD81 relative to mouse IgG_1_ isotype control (clone MOPC-31C, BD Pharmingen, San Diego, CA, anti-SR-BI and anti-E2 relative to human IgG (Southern Biotech, Birmingham, AL).

### Sera Collection and Inoculations

Eighy-six patients who underwent liver transplantation due to HCV infection and who presented recurrent hepatitis post-transplantation were selected. Patient blood was collected in Vacutainer® Rapid Serum Tube (Becton Dickinson, Franklin Lakes, NJ) and the serum was then separated after centrifugation at 1,000×g for 10 min. Serum was aliquoted and kept at −80°C. Viral load was determined by real-time PCR (COBAS TaqMan HCV Test, Roche Diagnostics, Mannheim, Germany). For inoculation of Huh-7.5/EG(4A/4B)GLuc cells, sera were thawed gently at 4°C. DMEM complete was aspirated from cells, which were washed 3x with PBS. Sera were diluted 1∶10, unless otherwise stated, to DMEM complete without FCS and the DMEM-sera mix was used to inoculate the cells, in duplicate. Four h post-inoculation, DMEM-sera mix was aspirated, cells were washed 3x with PBS, and then fed with DMEM complete with FCS for 120 h.

### In vitro Transcription, Electroporation of HCV RNAs, Generation of HCVcc Stocks, and Determination of Virus Titers in cell Culture Supernatants

These methods were employed as previously described [22].

### *Gaussia* Luciferase Assay

Cell culture supernatants from Huh-7.5/EG(4A/4B)GLuc cells were collected and centrifuged at 1,500×g for 10 min and cell-free supernatants were kept at 4°C until *Gaussia* luciferase measurements were taken. For each well, 2×50 µl supernatant was mixed with 50 µl *Gaussia* assay buffer (*Gaussia* Juice, PJK GmbH, Kleinblittersdorf, Germany) and then measured for 10 s in an Orion II Microplate Luminometer (Berthold Detection Systems, Pforzheim, Germany).

### HCV RNA Quantification by RT-qPCR

Viral RNA was isolated from HCVcc- or sera-inoculated cells and 25 ng of the total RNA sample was used for RT-quantitative PCR analysis with 5′ NTR-specific probe and primers as described in *Supporting Information S1*.

### Calculation of S/B, S/N, and Ź-factor

To validate the performance of the Huh-7.5/EG(4A/4B)GLuc cells for HTS assays, the signal-to-noise ratio (S/N), signal-to-background (S/B) and Ź-factor values were calculated using the methods described by Zhang *et. al.* [23]. In JC1 replication inhibition, Huh-7.5/EG(4A/4B)GLuc cells were seeded in 96 well plates, 1.2×10^4^ cells/well and treated with IFN-α (500 U/ml) or mock-treated as described above, followed by infection with JC1 viruses at an MOI 0.5 TCID_50_/cell. In JC1 entry inhibition, cells were seeded as described above. 1 h prior to infection cells were treated with anti-CD81 or mouse monoclonal control antibodies (both at 1 µg/ml). For each individual infection condition, 8 wells were used. At 5 days post infection, *Gaussia* activity was determined for each well in duplicate and S/B, S/N and Ź-factor values were calculated.

### Indirect Immunofluorescence

250 µl of electroporated cells (1×10^5^ cells) were seeded on glass coverslips in 24-well plates. After 72 h, cells were fixed with 4% paraformaldehyde in PBS and permeabilized with 0.5% Triton X-100 in PBS. In the case of patient sera or JC1 inoculations, cells were infected as described above, and fixed 96 h post inoculation. Immunostaining of NS5A protein was performed by using the mouse monoclonal 9E10 (kindly provided by Dr. C. Rice), of E2 protein by the anti-E2 human conformational mAb AR3A (kindly provided by Dr. M. Law) and of core by the mouse monoclonal anti-core antibody C7–50 (Santa Cruz Biotechnologies, Santa Cruz, CA), all at a final concentration of 1 µg/ml in PBS supplemented with 5% bovine serum albumin (BSA). Bound primary antibodies for the NS5A or the E2 were detected using goat α-mouse antibodies conjugated to AlexaFluor® 568 or goat α-human antibodies conjugated to AlexaFluor® 488 (Invitrogen, Eugene, OR), respectively, at a dilution of 1∶1,000 in PBS with 5% BSA. DNA was stained with DAPI (4′, 6-Diamidino-2-phenylindole dihydrochloride) (Sigma-Aldrich, St. Louis, MO). Finally, cells were washed 3x with PBS and once with water and mounted on glass slides with Fluoromount G (Southern Biotechnology Associates, Birmingham, AL).

### Quantification of HCV Positive Cells

To assess the number of HCV positive cells, cells seeded as described above were inoculated at various multiplicities of infection (MOI) for 4 h. Five days post infection, cells were fixed and the proportion of infected cells was monitored by NS5A-specific indirect immunofluorescence as described above. For each infection at least 1,000 cells were evaluated.

### Preparation of Cell Lysates, PAGE, and Western Blot Analysis

In order to prepare cell lysates for Western blot analysis, cells were lysed with 1% Triton X-100 in PBS. Proteins present in the cell lysates were separated by SDS-PAGE electrophoresis and transferred to a PVDF membrane for claudin, occludin, NS3, core, GFP, β-actin or GAPDH detection. A detailed description of this method is provided in *Supporting Information S1*.

### Quantification of HCV Core Protein

HCV core protein in cell culture supernatants was quantified using the Ortho® HCV antigen ELISA test kit (Ortho Clinical Diagnostics, Tokyo, Japan). Cell culture supernatants were harvested 120 h post infection, filtered through 0.45-µm-pore-size filters and kept at −80°C until the day of measurement. Colorimetric measurements were performed using a Sunrise colorimeter (Tecan Trading AG, Switzerland).

### Statistical Analyses

Data are presented as mean ± standard deviation (SD). The statistical comparison between two groups was made by a Mann-Whitney test. *p* value < 0.05 was considered to indicate a significant difference.

## Results

### Generation of the Huh-7.5/EG(4A/4B)GLuc Cell Line and Comparison with the Parental Huh-7.5 Cells

**Figure 1:**
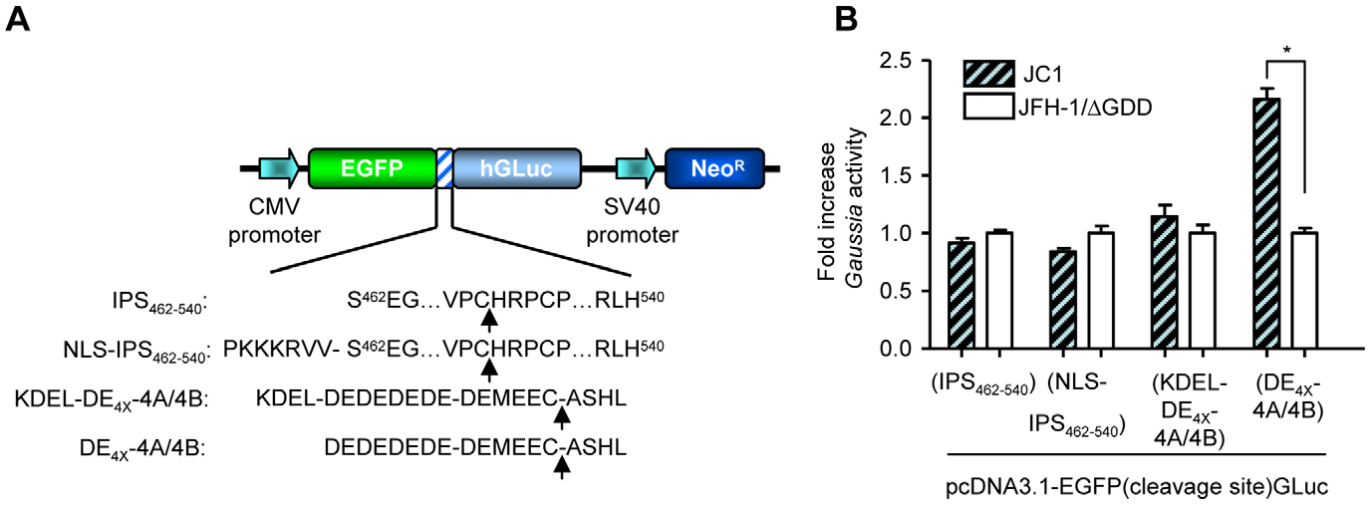
Schematic of dual-function reporter vectors used in the HCV NS3/4A protease activity assay. (A) The various recognition sites of NS3/4A protease, IPS_462–540_, NLS-IPS_462–540_, KDEL-DE_4x_-4A/4B and DE_4x_-4A/4B were inserted between the *EGFP* and the humanized *Gaussia luciferase* gene by an in-frame fusion. Arrows indicate the NS3/4A cleavage site. Expression of the reporter genes is under the human cytomegalovirus promoter (CMV) and the selection marker for the generation of the stable cell line is neomycin phosphotrasferase (Neo^R^). SV40, simian virus 40. (B) Transiently trasfected Huh-7.5 cells were infected with JC1 virus at an MOI 0.5 TCID_50_/cell or mock infected with JFH-1/ΔGDD supernatant. At 5 days post infection, the medium was harvested and *Gaussia* activity was measured. Results are expressed as the mean values from duplicate wells, measured in duplicates, from a representative experiment of 3 (mean ± SD; n = 4).*, *P*<0.05.

To generate a highly sensitive cell-based assay for HCV infectivity quantification, several reporter plasmids were constructed according to the NS3/4A-based reporter assay systems mentioned in the *Introduction* section. In our constructs the secreted alkaline phosphatase (SEAP) was replaced by the humanized form of the *Gaussia* luciferase (hGLuc) fused in-frame to the enhanced green fluorescence protein (EGFP) via various recognition sequences for the viral NS3/4A protease (Fig. 1A): i. the C-terminal region of IPS-1, encompassing the NS3/4A recognition site and a mitochondrial targeting sequence (amino acids 462–540, IPS_452–540_) ii. similar to (i) with the addition of an SV40 nuclear localization sequence (NLS) between the EGFP and the IPS segment (NLS-IPS_462–540_) iii. the ER retention signal (defined by amino acid sequence "KDEL") followed by octapeptide DEDEDEDE and the HCV genotype 1b NS4A/4B substrate sequence DEMEEC-ASHL iv. similar to (iii) without the ER retention signal.

Initially, these reporter constructs were transiently transfected to Huh-7.5 cells to examine whether they could be efficiently cleaved by NS3/4A protease following HCV infection (JC1 chimeric virus, at an MOI 0.5 TCID_50_/cell). At 5 days post infection, cell culture media were harvested and analyzed for *Gaussia* activity. As shown in Fig. 1B, elevated *Gaussia* levels, relative to control infections, were observed in these media harvested from cells that were transfected with the EGFP(DE_4x_-4A/4B)GLuc construct. This construct was similar to that referred by Iro *et. al*. [18], although Pan *et. al*. [19], failed to detect elevated amounts of SEAP activity with the same construct in a transient transfection and infection assay. The lack of success with the other constructs, including the IPS constructs and the KDEL-DE_4x_-4A/4B cassette, suggests that the viral recognition sequence may not be accessible by the NS3/4A protease caused by different protein conformations when the various peptide sequences are inserted into the chimera fusion protein.

Next, Huh-7.5 cells were stably transfected with plasmids encoding the EGFP(DE_4x_-4A/4B)GLuc construct, selected for 4 weeks under the pressure of G418 antibiotic and finally sorted for high EGFP expression, thus generating the Huh-7.5/EG(4A/4B)GLuc cell line. Expression of the known HCV entry receptors CD81 [24], SR-BI [25], claudin [26] and occludin [27] in the Huh-7.5/EG(4A/4B)GLuc cell line proved equal to that of the parental Huh-7.5 cells (Fig. S1A–C). Similar entry profiles for both cells were confirmed with infections by HCVpp for various genotypes (Fig. S1D). HCV replication in the Huh-7.5/EG(4A/4B)GLuc cell line was assessed by: (i) electroporation of JFH-1 subgenomic replicons carrying the *Firefly* luciferase reporter (Fig. S2A); and by (ii) electroporation of wild-type JFH-1 and the intra- or inter-genotypic chimeric derivatives, JC1 and Con1/JFH-1, respectively [13] (Fig. S2B). Both methods demonstrated that HCV replicates to similar levels in both cell lines, as deduced by the degree of *Firefly* luciferase expression in the sub-genomic replicon assay and by the extent of NS5A and E2 expression in those cells transfected with full-length viruses.

### Determination of the Specificity of the Huh-7.5/EG(4A/4B)GLuc Cell-based System

**Figure 2:**
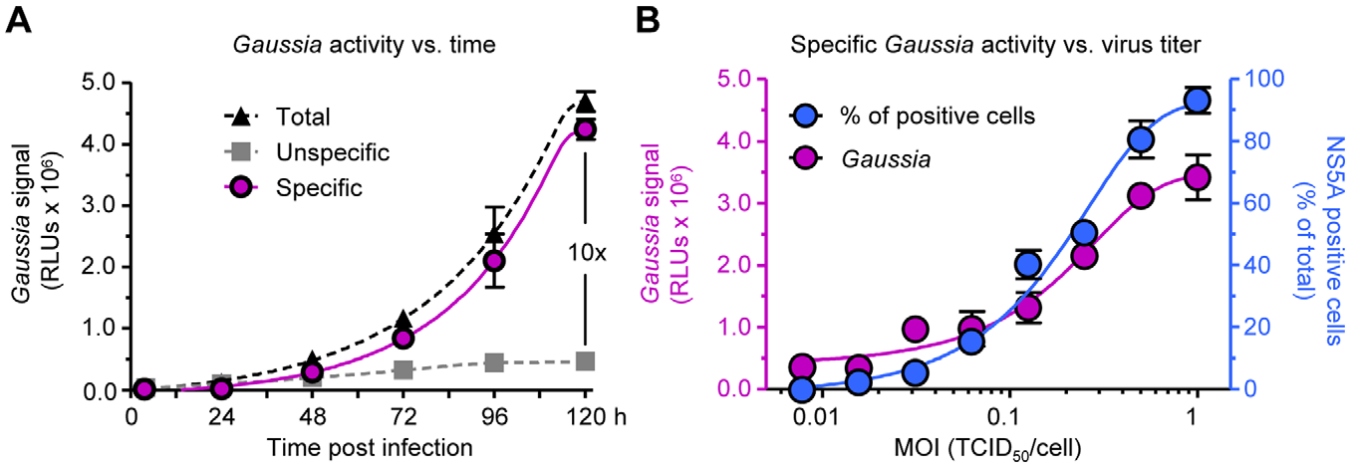
Specificity and sensitivity of the Huh-7.5/EG(4A/4B)GLuc reporter system. (A) Cells were infected with JC1 virus at an MOI of 0.5 TCID_50_/cell or mock (JFH-1/ΔGDD) infected. At the indicated time points, the *Gaussia* activity is given. (B) Cells were infected with JC1 virus at various MOIs (1, 0.5, 0.25, 0.125, 0.0625, 0.03125, 0.015625, 0.0 TCID_50_/ml) and the specific (total minus unspecific) *Gaussia* activity for each infection condition is given 5 days post-infection. In parallel, the quantification of the fraction of infected cells was performed by using indirect immunofluorescence (NS5A positive cells). Results are expressed as the mean values from duplicate wells, measured in duplicates, from a representative experiment of 3 (mean ± SD; n = 4).

To assess the specificity of the Huh-7.5/EG(4A/4B)GLuc cell line, cells were infected with JC1 virus at an MOI 0.5 TCID_50_/cell, and *Gaussia* activity was determined at different time points. Concomitantly, cells were infected with supernatant from JFH-1/ΔGDD-transfected cells as a negative control [22]. *Gaussia* activity acquired in JC1 infected-cells was considered total (specific and unspecific) while that acquired in the negative control was regarded as unspecific. Specific signal (total minus unspecific) increased over the specified time course and reached levels up to 10-fold higher than the unspecific signal 120 h post-infection (Fig. 2A). To determine the sensitivity of the Huh-7.5/EG(4A/4B)GLuc cell line and the number of infected cells that produce *Gaussia* signal at a given viral concentration, cells were infected with JC1 viruses at various MOIs; 120 h post infection the *Gaussia* activity as well as the number of NS5A positive cells were measured. As shown in Fig. 2B and Fig. S3, the maximum specific signal reached a plateau when MOIs ≥ 0.5 TCID_50_/cell were administered, which was corresponded to ≥75% of positive cells. For each point in Fig. 2B, any unspecific signal by a ΔGDD infection (≤10 %) was subtracted.

### Correlation of *Gaussia* Activity with HCV RNA and Protein Levels

**Figure 3:**
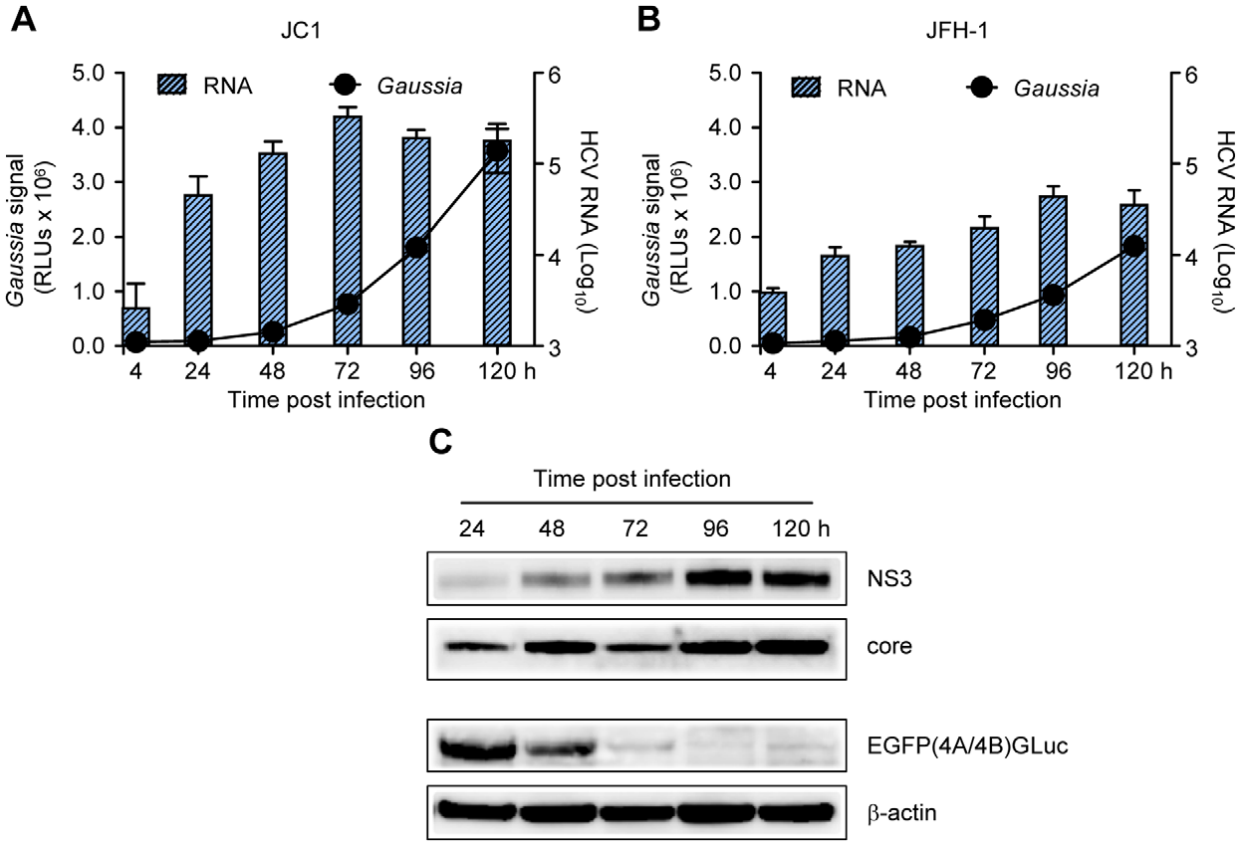
Correlation between extracellular *Gaussia* activity and HCV RNA and proteins. Huh-7.5/EG(4A/4B)GLuc cells were infected with JC1 (A) or JFH-1 (B) viruses at an MOI of 0.5 TCID_50_/cell. Extracellular *Gaussia* activity and intracellular RNA were analyzed at the given time points post infection. (C) Correlation between NS3 and core proteins levels with EG(4A/4B)GLuc cleavage efficiency. Huh-7.5/EG(4A/4B)GLuc cells were infected like in (A) and at the given time points expression levels of NS3, core and EG(4A/4B)GLuc were analyzed by Western blotting by anti-NS3, anti-core and anti-GFP antibodies, respectively.

To demonstrate that the *Gaussia* activity secreted by the Huh-7.5/EG(4A/4B)GLuc cells correlated with HCV RNA replication levels, the cell line was infected with JC1 or JFH-1 virus at an MOI 0.5 TCID_50_/cell. Subsequently, the *Gaussia* activity released in the medium and HCV RNA levels were determined side-by-side at different time points post-infection. As shown in Fig. 3A&B, viral RNAs peaked around 72 or 96 h post-infection for the JC1 or the JFH-1 infection respectively, and declined thereafter due to cellular confluence. However, a time-dependent increase in *Gaussia* activity occurred as a result of increasing NS3/4A protease amounts and *Gaussia* stability. This result implies that the *Gaussia* levels do not directly correlate with virus RNA replication levels. Notably, although equal virus inocula were used for JC1 and JFH-1 viruses, an approximately 2-fold higher RNA and *Gaussia* levels were detected in the JC1 virus analysis in comparison to JFH-1. Both viruses posses the JFH-1 RNA replication machinery (proteins NS3-NS5B) and therefore differences in RNA levels cannot be explained by higher replication capacity of the JC1 chimeric virus. Previously it has been shown that JC1 and JFH-1 virus replicate to the same extent in Huh7 and Huh7-derived clones [13]. However, JC1 virus produces a significantly higher amount of *in vitro* infectious virus at a more rapid kinetic manner and thus can spread faster in cell culture. Indeed, as shown in Fig. S4, at an MOI 0.1 TCID_50_/cell, JC1 virus spread was faster than that of JFH-1.

To correlate *Gaussia* activity with viral protein expression, the viral intracellular protein (core and NS3) levels and the amount of EGFP still associated with *Gaussia* protein, were determined post-infection with the JC1 virus at the same time points as previously. As shown in Fig. 3C, the cleavage induction between EG(4A/4B)GLuc correlated directly to increased NS3 protein levels. These data suggest that there needs to be a significant accumulation of NS3/4A post viral replication for efficient *Gaussia* cleavage and secretion which occurs at time points not earlier than 72 h post infection.

### Determination of Antiviral-mediated Neutralizations

**Figure 4:**
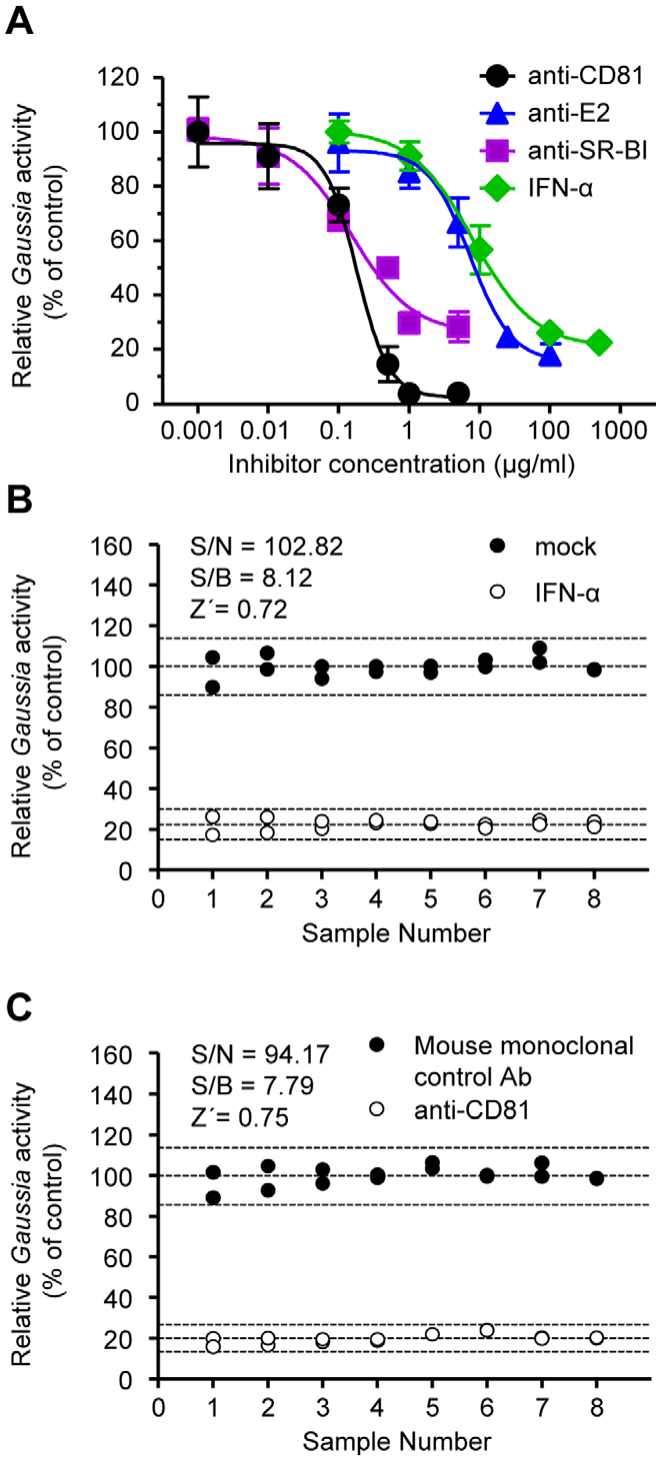
Quantification of HCVcc neutralization and estimates of statistical parameters of the screening system. (A) Huh-7.5/EG(4A/4B)GLuc cells were pre-incubated with the indicated doses of IFN-α, and anti-receptor antibodies (anti-CD81 or anti-SR-BI) or viruses were pre-incubated with anti-E2 antibodies prior to infections with JC1 virus (MOI 0.5 TCID_50_/cell). *Gaussia* specific activity was measured 5 days post-infection. The neutralizing activity is expressed as the percentage of inhibition, normalized to mock incubations. Results are from a representative experiment of three independent experiments. All points represent the mean of duplicate infections measured in duplicate (mean ± SD, n = 4). (B) Huh-7.5/EG(4A/4B)GLuc cells were pre-treated with IFN-α (open circles) or mock-treated (closed circles) for 18 h prior to infection with JC1 virus (MOI 0.5 TCID_50_/cell). 5 days post infection, *Gaussia* activity was measured and the statistical parameters S/N, S/B, and Ź-factor were estimated. (C) Huh-7.5/EG(4A/4B)GLuc cells were infected in the presence of anti-CD81 (open circles) or mouse monoclonal control antibodies (closed circles). 5 days post infection S/N, S/B, and Ź-factor were estimated similar to (B).

To determine the capability of the Huh-7.5/EG(4A/4B)GLuc cell line in antiviral-mediated neutralizations, cells were pre-treated with replication or entry inhibitors (IFN-α or anti-receptor antibodies [anti-CD81 or anti-SR-BI], respectively), or viruses were pre-incubated with the conformational anti-E2 antibody AR3A [28] prior to infection with JC1 virus at an MOI of 0.5 TCID_50_/cell. A clear dose-dependent inhibition in *Gaussia* secretion was acquired for all inhibitors (Fig. 4A). Specifically, the half maximal inhibitory concentrations (IC_50_) for the anti-CD81, anti-SR-BI, anti-E2 antibodies and IFN-α were 0.17, 0.38, 9.46 and 22.3 µg/ml, respectively.

Next, we assessed whether the Huh-7.5/EG(4A/4B)GLuc cells can be adopted for a high-throughput screening (HTS) assay for novel anti-HCV agents. For this validation, we calculated the signal to noise ratio (S/N), signal to background ratio (S/B) and the Ź-factor, in the presence or absence of IFN-α or anti-CD81 antibodies (replication or entry inhibitor, respectively), as described by Zhang *et. al.* [23]. As shown in Fig. 4B, the S/N and S/B ratio and the Ź-factor for IFN-α were 102.82, 8.12 and 0.72, respectively. Regarding the anti-CD81 antibodies (Fig. 4C), the S/N, S/B ratio and the Ź-factor were 94.17, 7.79 and 0.75, respectively.

### Screening of HCV Positive Sera with the Capacity for in vitro Replication

**Figure 5:**
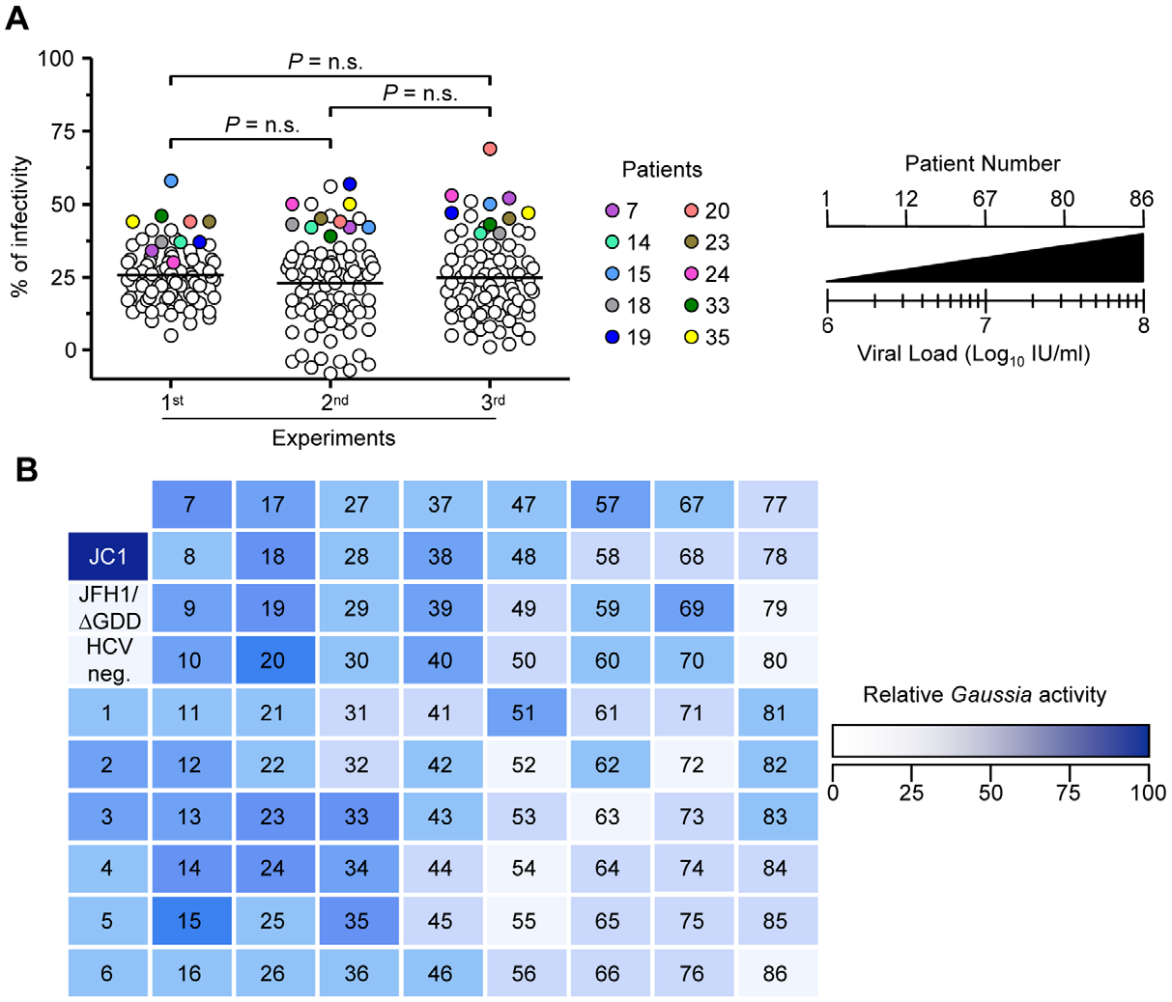
Overview of the screening strategy. (A) Eighty-six clinical sera were selected from patients as described in *Materials and Methods*. Patientś number rise relative to the viral load increase. Relative *Gaussia* expression scale: *Gaussia* activity by wells infected in parallel with the JC1 virus at an MOI of 0.5 TCID_50_/cell was set at 100 while that by JFH-1/ΔGDD and/or HCV control negative serum (HCV neg.) was set at 0. (A) Results from 3 representative experiments. Means difference between the experiments were not significant (n.s.). Patients with *Gaussia* activity ≥40% are indicated. (B) Colours were drawn according to the mean of 3 independent experiments. In each experiment duplicate infections were performed and measured in duplicate (n = 4).

We screened 86 sera derived from HCV genotype 1b-infected liver transplant recipients with respect to their *in vitro* replication potential by infecting the Huh-7.5/EG(4A/4B)GLuc cell line and deducing the *Gaussia* activity 5 d post-inoculation. The viral load in these samples ranged from 10^6^ to 10^8^ IU/ml. To facilitate the *Gaussia* readout, we set a relative scale of reporter activity; specifically, *Gaussia* obtained from cells that were in parallel infected with JC1 viruses was set to 100 while that obtained from mock-infected cells (JFH-1/ΔGDD infections) and/or HCV-negative sera (HCV neg.) was set to 0. Fig. 5A represents the results of three independent experiments while Fig. 5B represents the combined results of these experiments (means). Overall, *Gaussia* activity produced by cells infected with patient sera was low in comparison to that secreted by JC1 infected cells. Mean relative *Gaussia* activities for experiments 1, 2 and 3 were similar: 25.67 ± 1.035, 23.27 ± 1.561 and 24.80 ± 1.491, respectively (p = n.s.). Additionally, although *Gaussia* activity acquired from cells infected by few patients in one experiment was significantly higher than the mean, this result was not reproducible in the next experiment. Hence, Table 1 summarizes those patients whose *Gaussia* activity post infection of Huh-7.5/EG(4A/4B)GLuc cells was ≥ 40% and reproducible in 3 experiments (patients also indicated in Fig. 5A). Worth noting is the fact that the *in vitro* infection and replication of serum-derived HCV in Huh-7.5/EG(4A/4B)GLuc cells did not correlate with the initial HCV serum-viral load; in fact, *in vitro* infectivities for high-HCV-containing sera (10^7^–10^8 ^IU/ml, patient no. 67–86) were low.

**Table 1:** List of patients with infectivity ≥ 40%.

Patient Number	Viral Load (IU/ml×10^6^)	Infectivity^a^
7	2.58	42±10
14	3.40	40±3
15	3.41	50±8
18	3.82	40±3
19	3.93	47±10
20	4.14	52±14
23	4.33	44±1
24	4.45	46±9
33	5.00	43±4
35	5.00	47±3

a Infectivity is expressed as a percentage (% ± SD) relevant to that obtained from cells infected in parallel with JC1 viruses.

**Figure 6:**
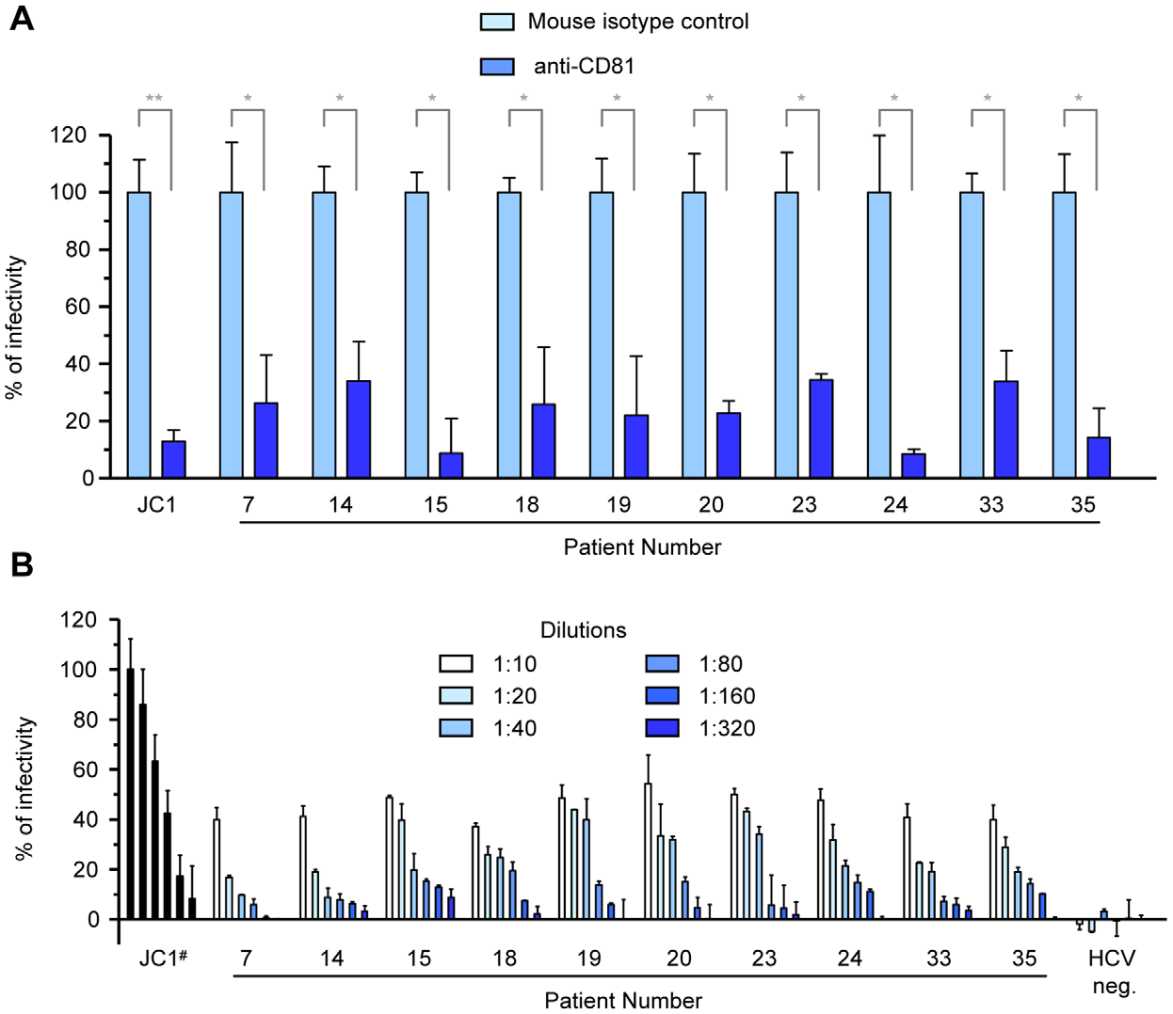
Specificity of serum infection by CD81-specific neutralizations and sera dilutions. (A) Huh-7.5/EG(4A/4B)GLuc cells were pre-incubated with CD81-specific (JS-81) antibodies or isotype-matched control antibodies for 1 h prior to infections. *Gaussia* activity in the anti-CD81 pre-incubated cells (dark blue bars) is expressed as percentage (%) relative to that obtained for the control infections. Control infections are represented as light blue bars. (B) Huh-7.5/EG(4A/4B)GLuc cells were inoculated with serial dilutions of JC1 viruses, patient sera or control HCV negative serum. 120 h post infection *Gaussia* activity in the supernatant of the infected cells was determined. Results for (A) and (B) are expressed as the mean values from duplicate wells, measured in duplicates, from a representative experiment of 3 (mean ± SD; n = 4). #Serial dilutions of JC1 virus: MOI at 1, 0.5, 0.25, 0.125, 0.0625 and 0.03125 TCID_50_/cell.

To prove the specificity of serum infections, Huh-7.5/EG(4A/4B)GLuc cells were pre-incubated with anti-CD81 antibodies prior to inoculations with the selected sera presented in Table 1. As shown in Fig. 6A, JC1 control as well as serum infections were potently inhibited by anti-CD81 antibodies. To further corroborate this specificity, cells were inoculated with serial dilutions of JC1 viruses or sera and the *Gaussia* activity was monitored 120 h post infection (Fig. 6B). As expected, the magnitude of *Gaussia* activity correlated well with the amount of inoculum, demonstrating that it reflects the infectious titer in a quantitative manner.

**Figure 7:**
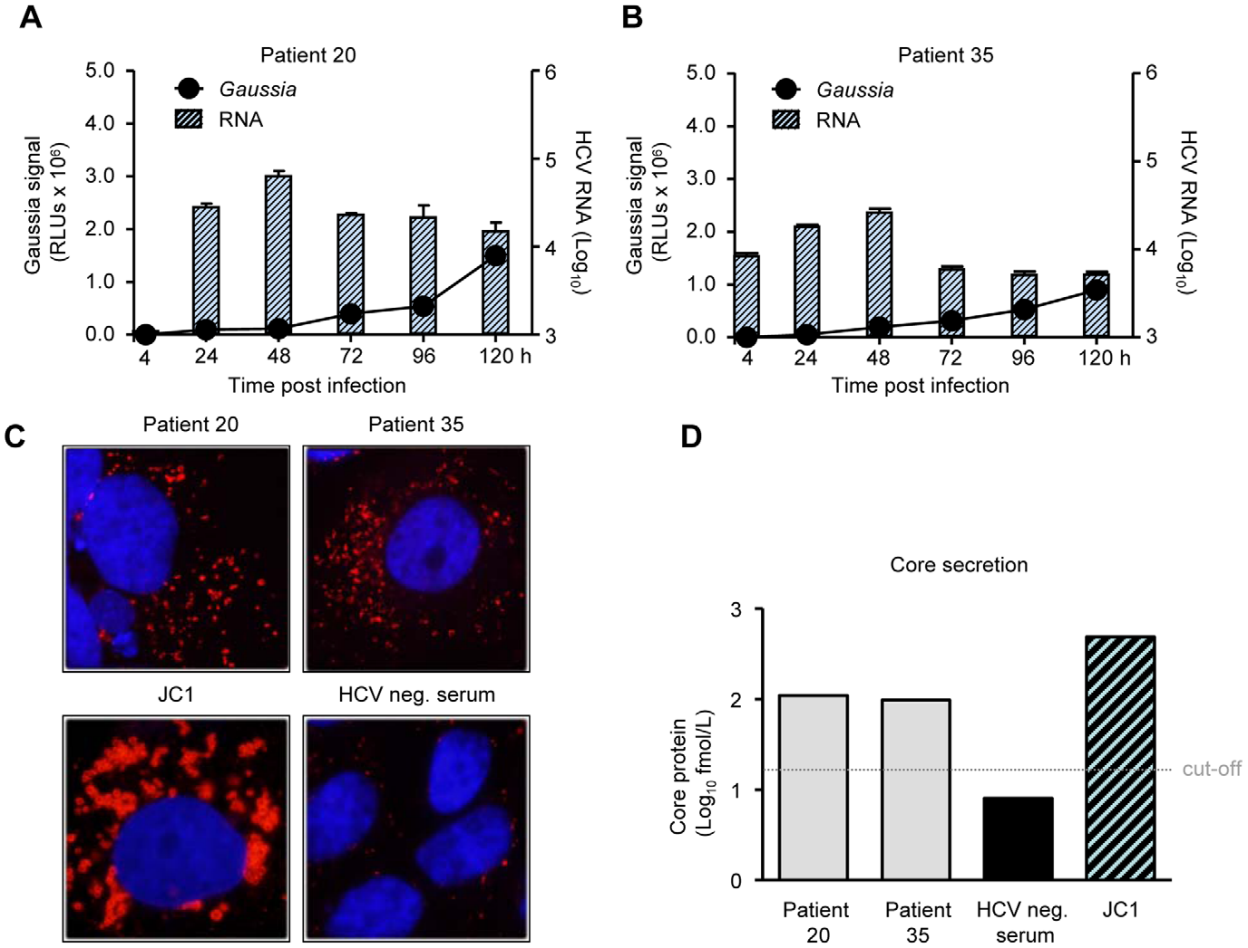
Productive replication of serum-derived HCV in Huh-7.5/EG(4A/4B)GLuc cells. (A&B) *Gaussia* activity and intracellular HCV RNA analysis in Huh-7.5/EG(4A/4B)GLuc cells inoculated with sera derived from patients 20 and 35 at the indicated time points. Results represent the mean values from duplicate wells, measured in duplicate (for both *Gaussia* and RNA), from a representative experiment of 3 (mean ± SD; n = 4) (C) Intracellular core expression in Huh-7.5/EG(4A/4B)GLuc cells inoculated with the indicated patient sera, JC1 virus or HCV negative Serum (HCV neg.) as negative control, as detected by core protein IF. Cells were stained with α-core specific antibodies (clone C7–50) and α-mouse Alexa-568 antibodies (red). Cell nuclei were counterstained with DAPI (blue), magnification 100x. (D) Core protein released in the supernatant from Huh-7.5/EG(4A/4B)GLuc cells infected by the indicated patient sera, JC1 virus or HCV neg. serum as negative control, 120 h post inoculation.

To demonstrate that serum-derived HCV did replicate productively in Huh-7.5/EG(4A/4B)GLuc cells, HCV RNA and *Gaussia* activity were analyzed at different time points post-inoculation for 2 patients. As shown in Fig. 7A&B, both intracellular HCV RNA and extracellular *Gaussia* activity kinetics were similar to those obtained by HCVcc infections, indicating a productive *in vitro* replication of serum-derived HCV. Furthermore, immunofluorescence (IF) staining of capsid (core) protein in inoculated cells was performed (Fig. 7C). Core positive cells for JC1 infected cells were detectable as early as 24 h post infection whereas core positive cells for serum-inoculated cells were present from 72 h post inoculation and thereafter. IF staining 96 h post inoculation was substantially more intense in the JC1 infected cells. The less intense core signal in serum-inoculated rather reflects less RNA replication as deduced by the RT-qPCR data than antigenic differences by the anti-core antibody or distinct half-life times of the core molecules. Finally, extracellular core was measured by core ELISA 120 h post inoculation (Fig. 7D). Huh-7.5/EG(4A/4B)GLuc cells infected with JC1 viruses presented a significant core release (∼500 fmol/l). Interestingly, cells infected with patient sera did release measurable core amounts in the supernatant (∼100 fmol/l) suggesting that complete HCV replication took place in Huh-7.5/EG(4A/4B)GLuc cells post inoculation with the patient sera.

## Discussion

In the present study we established a robust *Gaussia* luciferase cell line, designated Huh-7.5/EG(4A/4B)GLuc, which is as permissive as the parental Huh-7.5 cells in terms of HCV infection and in enabling the rapid and accurate quantification of HCV infections in cell culture via JFH-1 wild-type- or JFH-1-derived viruses. We determined the secretion of *Gaussia* reporter activity based on the HCV NS3/4A protease activity, as has been previously described in other NS3/4A-dependent systems [18], [20], [29].

Initially, several engineered *Gaussia* fusion proteins were constructed with diverse NS3/4A-cleavable peptides of NS3/4A protease. Out of the 4 constructs examined, only the EGFP(DE_4x_-4A/4B)Gluc was functional (Fig. 1). Pan *et. al*., while developing their similar dual reporter cell line Huh-7.5/EG(Δ4B5A)SEAP [19], checked various cleavable peptides as well, including the 4A/4B signal sequence utilized in this study. In an EGFP-SEAP configuration, the 4A/4B cleavage site appeared to be inaccessible by the viral protease. Indeed, Iro *et. al*. [18], demonstrated that the insertion of the octapeptide DEDEDEDE prior to the NS4A/4B recognition sequence is crucial for a cleavage efficiency enhancement, which appeared also true for our EGFP-*Gaussia* configuration. Interestingly, we established Huh-7.5/EGFP-IPS and Huh-7.5/EGFP-NLS-IPS cells lines, similar to Jones *et. al.* [20]; in these cells, upon JC1 infection, we observed EGFP re-localization in the whole cytoplasm or in the nucleus, respectively (data not shown). Nonetheless, C-terminal *Gaussia* gene addition to both EGFP-IPS chimeric configurations appeared ineffective with respect to specific *Gaussia* secretion upon JC1 infection, likely due to cleavage interference caused by different protein conformations.

Our *Gaussia*-based reporter system appears a better alternative than the previously reported SEAP cell lines. Indeed, the Huh-7.5/EG(4A/4B)GLuc cell line combines the advantages of the previously reported Huh7-J20 and Huh-7.5/EG(Δ4B5A)SEAP cell lines while also retaining the unique features of the *Gaussia* reporter. These specialized features can be enumerated: (i) as *Gaussia* is naturally secreted into the media, no cell lysis is required for the detection of its enzymatic activity; (ii) the secreted indicator function not only permits multiple kinetics experiments using only one culture through the sequential sampling of the medium, but also allows the cells to be used for other purposes, such as RNA extraction, Western blot analysis, and other assays; (iii) *Gaussia* is non-toxic and very potent - compared to the humanized forms of firefly (hFLuc) and Renilla (hRLuc) luciferases expressed under similar conditions, the humanized form of *Gaussia* (hGLuc) generates over a 1000-fold higher bioluminescent signal intensity from live cells (in concert with their immediate environment) and over a 100-fold higher intensity from viable cells alone (not including secreted luciferase) or cell lysates [21]; (iv) *Gaussia* is stable in culture medium (with a half-life ∼6 d) [30] and samples can thus be stored at 4°C for several days without any significant change in its activity levels; (v) it is HCV genotype-independent, and therefore does not require any genetic modification of the viral genomes, due to the highly conserved nature of the NS3/4A recognition sequence [31].

Characterization of the Huh-7.5/EG(4A/4B)GLuc cell line demonstrated that *Gaussia* activity in the culture medium correlated precisely with the virus inoculum (Fig. 2B). In order to obtain maximum *Gaussia* sensitivity, however, virus inoculations should be performed with MOI ≥ 0.5 TCID_50_/cell, although accurate infection quantifications were possible with MOIs as low as ≤ 0.1 TCID_50_/ml. This unique characteristic suggests that the Huh-7.5/EG(4A/4B)GLuc reporter system could be utilized in studies where small or slowly replicating virus samples could applied; e.g., in the screening of patient sera for the presence of *in vitro* replication-competent isolates. In addition, we also showed that a 5-day infection assay is the optimal time condition for achieving significant increased amounts of *Gaussia* activity in culture medium (Fig. 2A) although already at day 4 post-infection *Gaussia* activity was detectable. This time-frame brings our assay at the same position like that presented by Iro *et. al*., and 1 day faster than that presented by Pan *et. al*. In contrast, Jones *et. al.*, were able to monitor HCV infection by fluorescence re-localization in their Huh-7.5 cells expressing RFP-NLS-IPS construct as early as 10–12 h post infection. This fluorescence system appeals ideal for live cell microscopy at a single-cell level, which makes it attractive for elucidation of viral and cellular mechanisms during HCV life cycle. Whether that system could be applied for HTS assays needs to be addressed; microscopy-based HTS have been already described, though with the demand of costly microscopy apparatus and computer software.

*Gaussia* read from Huh-7.5/EG(4A/4B)GLuc cells infected in the presence of selected inhibitors (IFN-α or entry inhibitors) demonstrated the potential use of this reporter system for HTS of entry inhibitors and other antiviral compounds. Estimates of statistical parameters for a potential screening system exist only in the study of Pan *et. al*. In our system, the S/N, S/B ratio and the Ź-factor ranged between 94.17–102.82, 7.79–8.12 and 0.72–0.75, respectively, clearly superior to the SEAP reporter cell line whose these parameters ranged between 21.2–34–1, 3.8–3.9 and 0.64–0.74, respectively.

Since the discovery of HCV in 1989, several researchers have tried to inoculate cells *in vitro* using serum-derived HCV. These efforts involved liver cells as well as cells of non-liver origin including HeLa, CEM, H9, Jurkat, Molt 3, Molt 4, U937, P3HR1, Raji and Daudi cells [32]. Although the JFH-1 isolate can replicate in hepatoma cell lines of human and murine origin [33], high replication levels can only be achieved in select subclones of the human hepatoma cell line Huh-7, which are highly permissive for HCV replication; e.g., Huh-7.5 [9] or Lunet cells [10]. These cell lines harboured a subgenomic replicon and were “cured” by IFN-α or a polymerase inhibitor, thereby preserving the high replication capacity observed in HCV.

In this study, we provide evidence for a productive infection and replication of the Huh-7.5/EG(4A/4B)GLuc cells by clinical sera derived by patients who underwent liver transplantation due to HCV infection and who presented recurrent hepatitis post-transplantation. Studies on HCV kinetics during and immediately after LT have shown that, although a rapid drop of HCV viral load occurs within the initial hours after liver graft reperfusion, the viral load increases as soon as 12 h after graft reperfusion exceeding pre-transplantation levels by 1 to 3 months after transplantation in a significant portion of HCV patients who undergo liver transplantation [34]. Here, we chose eighty-six sera harvested 3–6 months post LT from patients with hepatitis C recurrence. In the transplant setting however, the anti-HCV response takes place (in most cases) in the context of a nonself histocompatibility complex, in addition to strong immunosupression [35]. Additionally, viral load values in transplant patients exceed pre-LT values by 0.96 log IU/ml [36]. These data argue for a LT patient-sera selection against sera derived by chronic patients. Indeed, ∼12% of those LT-patient sera contained *in vitro* replication-competent viruses, as deduced by the *Gaussia* signal, real time quantitative PCR, immunofluorescence and capsid protein secretion. Nevertheless, this infection was less efficient than that with recombinant HCV virus produced in cell culture.

Since the initial discovery of the JFH-1 virus and its extraordinary capacity to support the complete HCV life cycle in vitro several JFH-1-derived chimeric virus with other isolates have been described [12], [13], [14], [37], [38]. Common characteristic of these chimeric viruses (except the chimeric virus JC1) is the requirement of cell culture adaptive mutations that increase virus titers without affecting replication. This finding was also true for the initial JFH-1 genome [39], [40]. Our RNA and *Gaussia* kinetic analyses for JC1, JFH-1 and sera-inoculated cells (Fig. 2 and 6) suggest that although natural viruses contained in patient sera were able to infect Huh-7.5/EG(4A/4B)GLuc cells and propagate *in vitro*, their replication and spread kinetics are less efficient than those obtained with the highly *in vitro* adapted JC1 virus. Sera-derived HCV appears a weakness rather for *in vitro* replication than entry into Huh-7.5 cells (G. Koutsoudakis and X. Forns, unpublished data) which may cause the major contribution for the *in vitro* propagation incompetence. However, our results presented in Fig. 6 & 7 clearly show a productive infection of Huh-7.5/EG(4A/4B)GLuc cells by patient sera.

Furthermore, although our extensive efforts to minimize inter-assay variation which could have been produced by manipulations of the sera (e.g. multiple freeze-and-thaw cycles) or the *Gaussia* assay itself (e.g. maintain the same source of *Gaussia* substrate), the results obtained by a single serum infection may vary at a higher level in comparison to JC1 infections. Indeed, JC1 virus is the product of a unique clone and it was always prepared fresh; clinical sera contain a variety of infectious and non-infectious quasispecies which could produce some extra variation among experiments. Therefore, in order to confirm our infection results produced by the initial screening (Fig. 5), in the subsequent experiments (Fig. 6 & 7) we chose only those sera with the highest infection capacity accompanied by high inter-assay reproducibility. Our efforts at the moment concentrate on testing the infectious progeny release in infection experiments or whether these patient-derived viruses could establish persistent infection in cell culture. Assuming that we could be successful in these efforts, we do believe that we should proceed in cloning new recombinant HCV isolates which may also posses cell culture adaptive mutations.

Altogether, we believe that our robust, specific, and sensitive *Gaussia* cell-based assay, utilizing the Huh-7.5/EG(4A/4B)GLuc cell line, will facilitate the further screening of *in vitro* replication-competent serum-derived viruses, as well as the subsequent cloning of recombinant HCV isolates. In addition, we believe it will prove effective for the HTS of entry inhibitors and other antiviral compounds.

## Supporting Information

Figure S1 **HCV receptor and HCVpp entry analyses in Huh-7.5 and Huh-7.5/EG(4A/4B)GLuc cells.** (A & B) Expression of SR-BI and CD81 on the surfaces of Huh-7.5 or Huh-7.5/EG(4A/4B)GLuc cells, respectively. (C) Occludin and claudin expression as determined by Western blot. (D) Infectivity of Huh-7.5 *vs.* Huh-7.5/EG(4A/4B)GLuc cells with HCVpp. Results are expressed as the mean values from duplicate wells, measured in duplicates from a representative experiment of 3 (mean ± SD; n = 4).
(TIF)

Figure S2 **HCV replication analysis in Huh-7.5 and Huh-7.5/EG(4A/4B)GLuc cells.** (A) *Firefly* luciferase activity in Huh-7.5 *vs.* Huh-7.5/EG(4A/4B)GLuc electroporated cells with the subgenomic replicon RNAs carrying *Firefly* luciferase as a reporter. Results are expressed as the mean values from duplicate wells, measured in triplicate, from a representative experiment of 3 (mean ± SD; n = 6). (B) Immunofluorescence analysis of Huh-7.5/EG(4A/4B)GLuc cells electroporated with JC1 (panels *I* to *IV*), Con1/JFH-1 (panels *V* to *VIII*), or JFH-1 (panels *IX* to *XII*) or Huh-7.5 cells electroporated with JC1 (panels *XIII to XIV*, positive control). Cells were stained with anti-E2 specific antibodies or anti-NS5A specific antibodies and anti-human Alexa-468 (green) or anti-mouse Alexa-568 (red) antibodies, respectively. Cell nuclei were counterstained with DAPI (blue), magnification 100x.
(TIF)

Figure S3 **Immunofluorescence analysis of Huh-7.5/EG(4A/4B)GLuc cells 120 h post infection with JC1 virus.** Huh-7.5/EG(4A/4B)GLuc cells were infected with JC1 virus at various MOIs as indicated on each image. Cells were stained with anti-NS5A specific antibodies and anti-mouse Alexa-568 (red) antibodies. Cell nuclei were counterstained with DAPI (blue), magnification 20x.
(TIF)

Figure S4 **Immunofluorescence analysis of Huh-7.5/EG(4A/4B)GLuc cells infected with JC1 or JFH-1 viruses at an MOI 0.1 TCID_50_/cell.** Cells were infected with JC1 or JFH-1 viruses and at the indicated time points post infection cells were stained with anti-NS5A specific antibodies and anti-mouse Alexa-568 (red) antibodies. Cell nuclei were counterstained with DAPI (blue), magnification 10x.
(TIF)

Supporting Information S1 **Protocols for plasmid construction and establishment of the** ***Gaussia*** **cell line Huh-7.5/EG(4A/4B)GLuc, HCVpp production and infection, flow cytometry, transient HCV replication using** ***Firefly*** **luciferase reporter genomes, HCV RNA quantification by RT-qPCR and preparation of cell lysates, PAGE, and Western blot analysis are provided in this section.**
(DOC)
