# Supplementary figures and images for: A Gaussia Luciferase Cell-Based System to Assess the Infection of Cell Culture- and Serum-Derived Hepatitis C Virus
Source: PLoS One. 2012 Dec 31;7(12):e53254. doi: 10.1371/journal.pone.0053254 (PMC3534054; doi:10.1371/journal.pone.0053254)

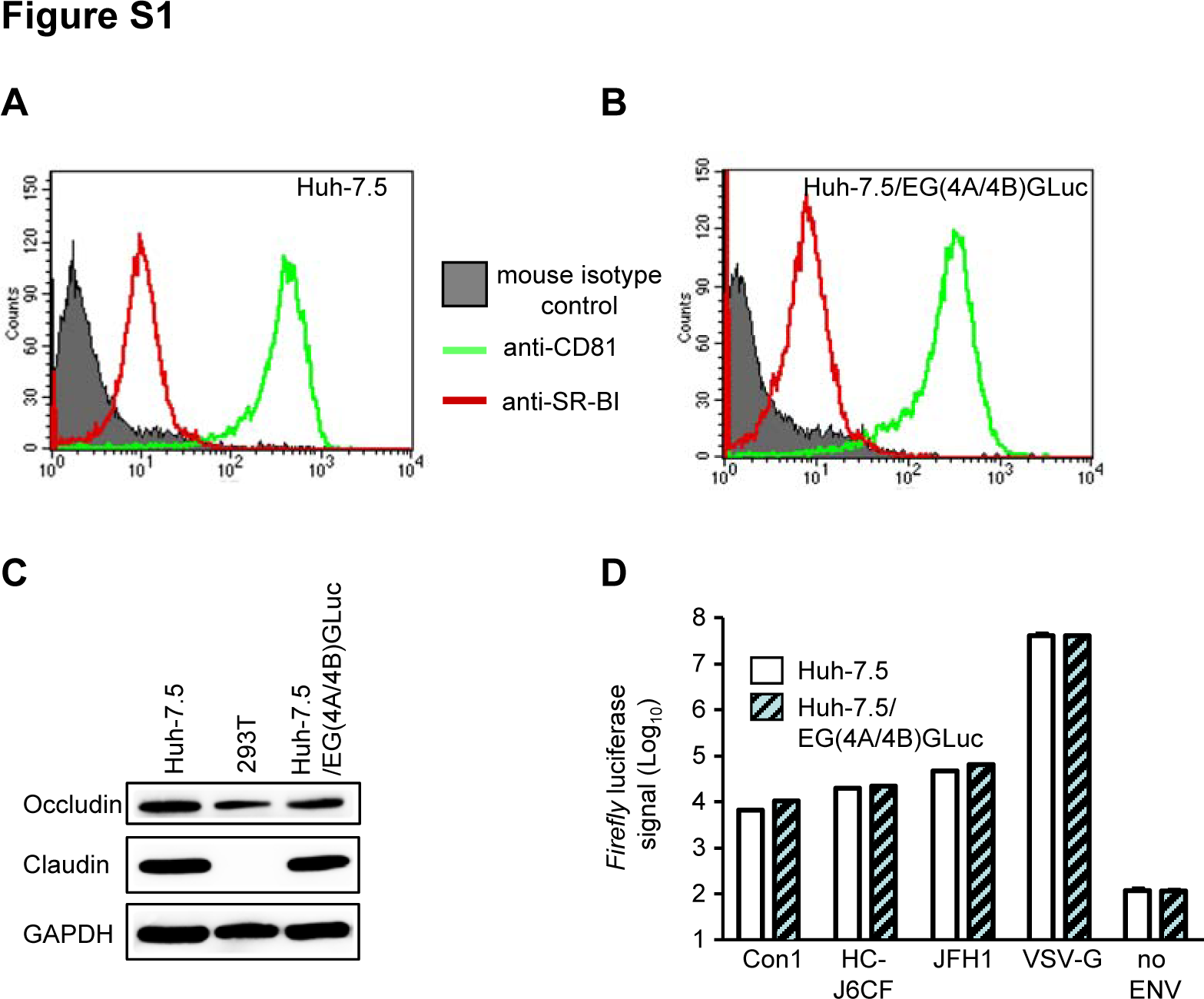

Supplement: Figure S1 — HCV receptor and HCVpp entry analyses in Huh-7.5 and Huh-7.5/EG(4A/4B)GLuc cells. (A & B) Expression of SR-BI and CD81 on the surfaces of Huh-7.5 or Huh-7.5/EG(4A/4B)GLuc cells, respectively. (C) Occludin and claudin expression as determined by Western blot. (D) Infectivity of Huh-7.5 vs. Huh-7.5/EG(4A/4B)GLuc cells with HCVpp. Results are expressed as the mean values from duplicate wells, measured in duplicates from a representative experiment of 3 (mean ± SD; n = 4). (TIF) [file pone.0053254.s001.tif]

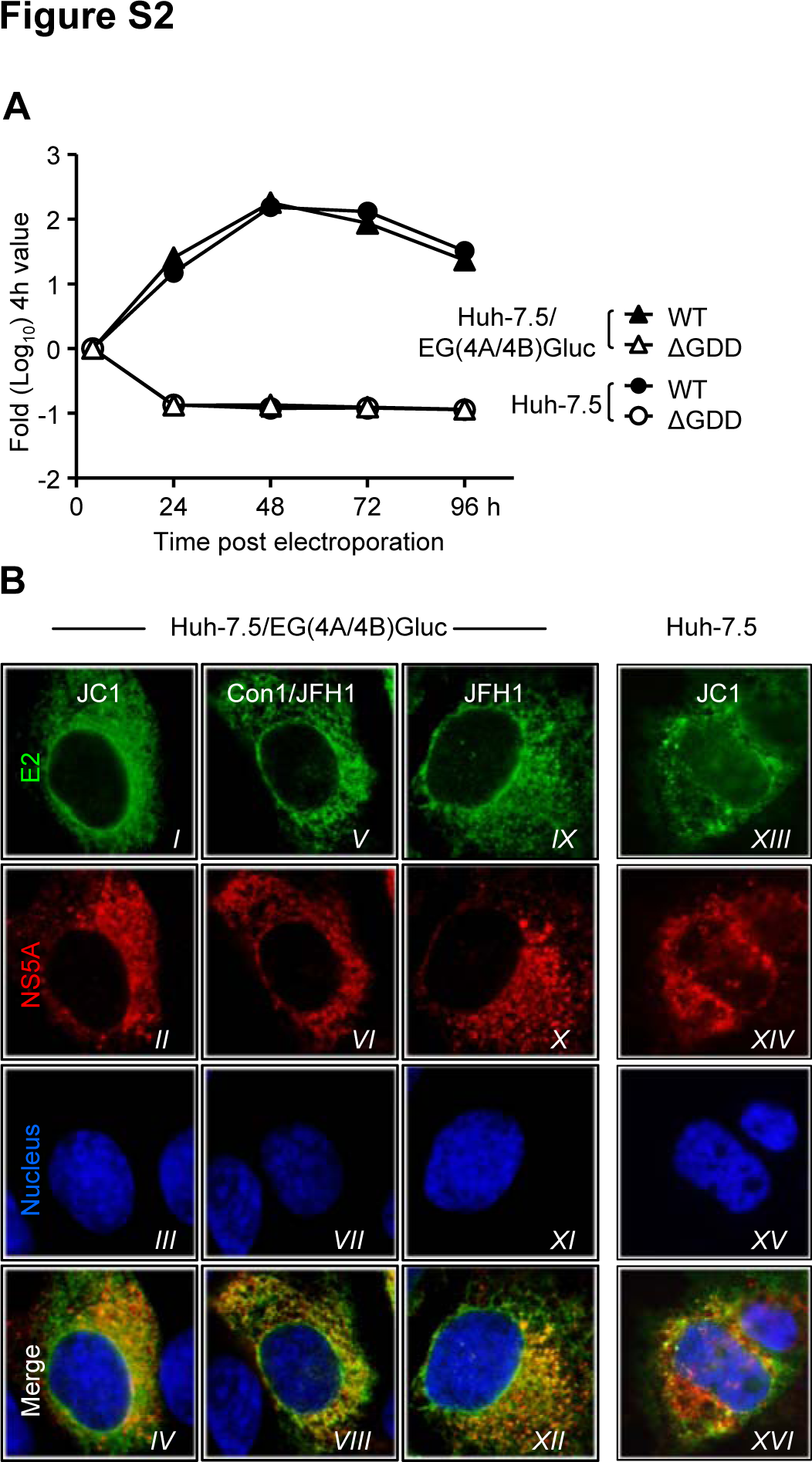

Supplement: Figure S2 — HCV replication analysis in Huh-7.5 and Huh-7.5/EG(4A/4B)GLuc cells. (A) Firefly luciferase activity in Huh-7.5 vs. Huh-7.5/EG(4A/4B)GLuc electroporated cells with the subgenomic replicon RNAs carrying Firefly luciferase as a reporter. Results are expressed as the mean values from duplicate wells, measured in triplicate, from a representative experiment of 3 (mean ± SD; n = 6). (B) Immunofluorescence analysis of Huh-7.5/EG(4A/4B)GLuc cells electroporated with JC1 (panels I to IV), Con1/JFH-1 (panels V to VIII), or JFH-1 (panels IX to XII) or Huh-7.5 cells electroporated with JC1 (panels XIII to XIV, positive control). Cells were stained with anti-E2 specific antibodies or anti-NS5A specific antibodies and anti-human Alexa-468 (green) or anti-mouse Alexa-568 (red) antibodies, respectively. Cell nuclei were counterstained with DAPI (blue), magnification 100x. (TIF) [file pone.0053254.s002.tif]

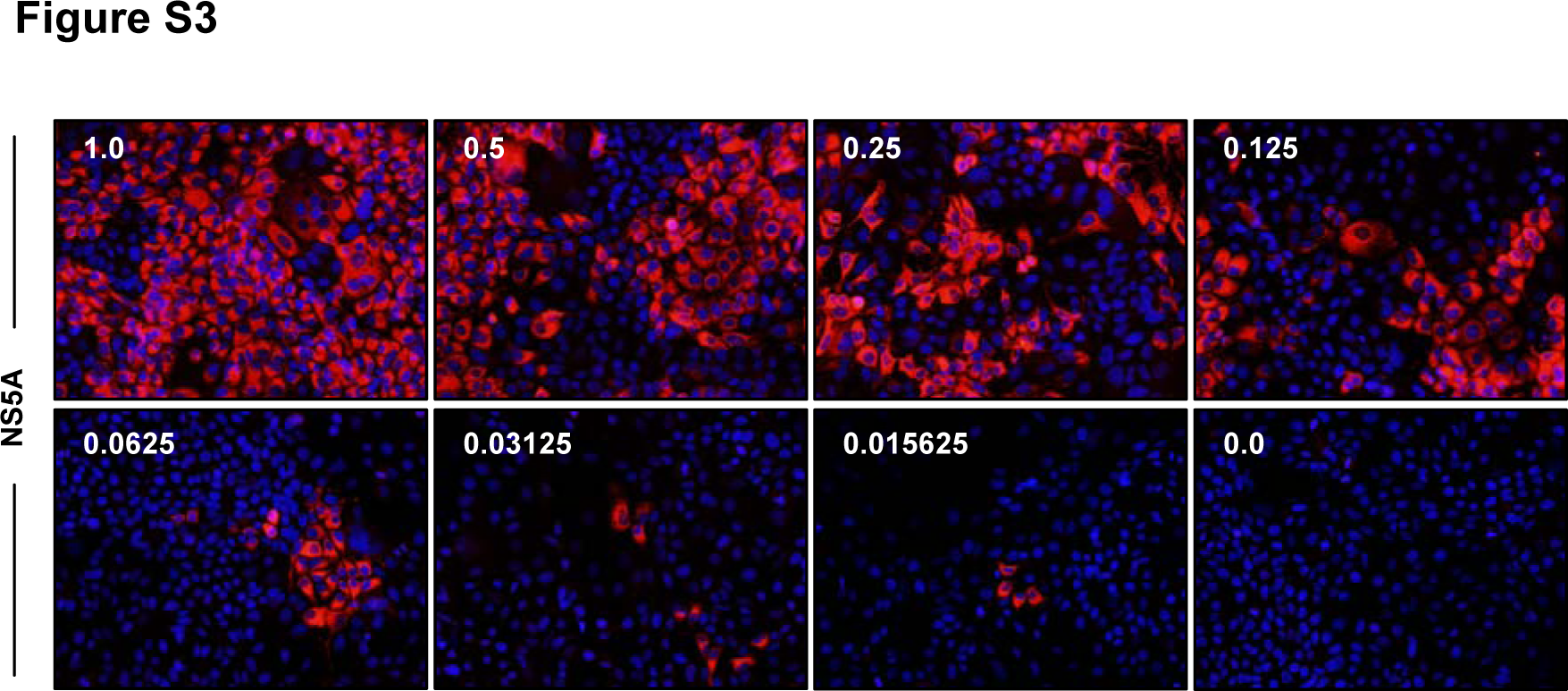

Supplement: Figure S3 — Immunofluorescence analysis of Huh-7.5/EG(4A/4B)GLuc cells 120 h post infection with JC1 virus. Huh-7.5/EG(4A/4B)GLuc cells were infected with JC1 virus at various MOIs as indicated on each image. Cells were stained with anti-NS5A specific antibodies and anti-mouse Alexa-568 (red) antibodies. Cell nuclei were counterstained with DAPI (blue), magnification 20x. (TIF) [file pone.0053254.s003.tif]

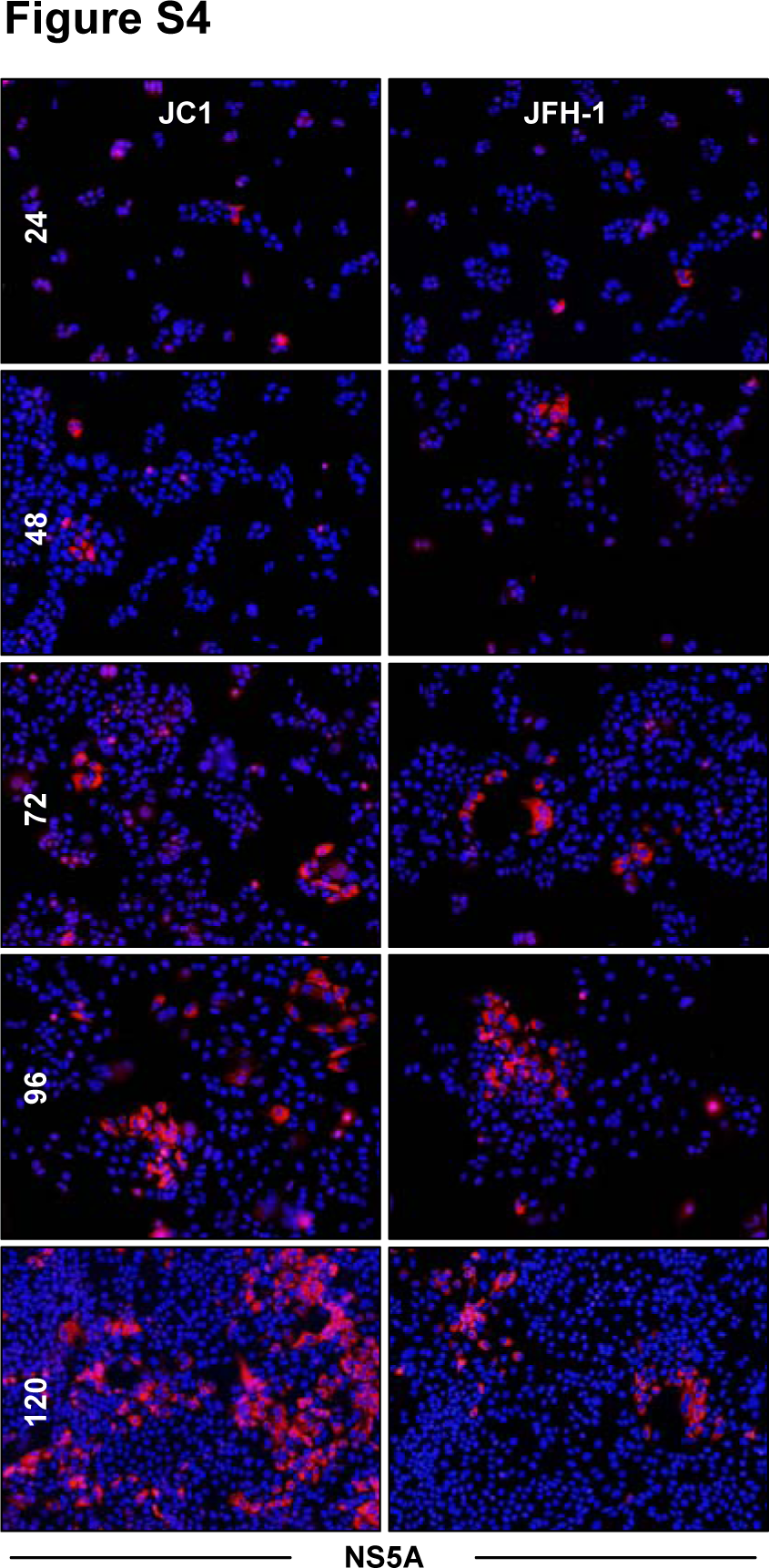

Supplement: Figure S4 — Immunofluorescence analysis of Huh-7.5/EG(4A/4B)GLuc cells infected with JC1 or JFH-1 viruses at an MOI 0.1 TCID50/cell. Cells were infected with JC1 or JFH-1 viruses and at the indicated time points post infection cells were stained with anti-NS5A specific antibodies and anti-mouse Alexa-568 (red) antibodies. Cell nuclei were counterstained with DAPI (blue), magnification 10x. (TIF) [file pone.0053254.s004.tif]
