# Supplementary material for: A Gaussia Luciferase Cell-Based System to Assess the Infection of Cell Culture- and Serum-Derived Hepatitis C Virus
Source: PLoS One. 2012 Dec 31;7(12):e53254. doi: 10.1371/journal.pone.0053254 (PMC3534054; doi:10.1371/journal.pone.0053254)
Supplement: Supporting Information S1 — Protocols for plasmid construction and establishment of the Gaussia cell line Huh-7.5/EG(4A/4B)GLuc, HCVpp production and infection, flow cytometry, transient HCV replication using Firefly luciferase reporter genomes, HCV RNA quantification by RT-qPCR and preparation of cell lysates, PAGE, and Western blot analysis are provided in this section. (DOC) [file pone.0053254.s005.doc]

# Supporting Information S1

**A *Gaussia* luciferase cell-based system to assess the infection of cell culture- and serum-derived hepatitis C virus**

George Koutsoudakis^1^, Sofía Pérez-del-Pulgar^1^, Patricia González^1^, Gonzalo Crespo^1^, Miquel Navasa^1^, Xavier Forns^1^

^1^ Liver Unit, Institut D'Investigacions Biomèdics August Pi i Sunyer, Centro de Investigación Biomédica en Red: Enfermedades Hepáticas y Digestivas, Hospital Clínic, Barcelona, Spain

## Supporting Material and Methods

## Plasmid construction and establishment of the Gaussia cell line Huh-7.5/EG(4A/4B)GLuc

The plasmids pcDNA3.1/EG(DE_4x_-4A/4B)GLuc and pcDNA3.1/EG(KDEL-DE_4x_-4A/4B)GLuc encoding EGFP fused in-frame with the octapeptide DEDEDEDE (DE_4x_) or the ER retention signal KDEL and the octapeptide DE_4x_ respectively, followed by the NS4A/4B protease recognition sequence DEMEEC-ASHL and the humanized gene of the *Gaussia* luciferase were assembled by PCR amplification as follows: the EGFP gene was PCR amplified with the primers F-GFP-BamHI (5´-GCTCGGATCCGCCACCATGGTGAGCAAGGGCGAGGAGCT-3´) and R-GFP-EcoRI (5´-TGCAGAATTCTTACTTGTACAGCTCGTCCATGCCGAGA-3´), cut with the restriction enzymes BamHI and EcoRI, and then inserted into the pcDNA3.1 vector (Invitrogen, Eugene, OR), thereby generating the plasmid pcDNA3.1-EGFP. The octapeptide DE_4x_ or the ER retention signal KDEL and the octapeptide DE_4x_ followed by the NS4A/4B protease recognition sequence DEMEEC-ASHL and the humanized gene of the *Gaussia luciferase* were amplified in one PCR reaction with the primers S-DEDE-GL (5´-CGAGCTGTACAAGGACGAGGACGAGGACGAGGACGAGGATG AGATGGAAGAGTGCGCCTCACACCTCATGGGAGTCAAAGTTCTGTTTGCCCTGATCT-3´) or S-KDEL-DEDE-GL (5´-CGAGCTGTACAAGACGAGCTGGACGA GGACGAGGACGAGGACGAGGATGAGATGGAAGAGTGCGCCTCACACCTCTGGGAGTCAAAGTTCTGTTTGCCCTGATCT-3´) and A-GL-EcoRI (5´- GCTGGATATCTGCAGAATTCTTAGTCACCACCGGCCCCCTTGATCTTGTC-3´), cut with the restriction enzymes BsrGI and EcoRI, and then inserted into the pcDNA3.1-EGFP plasmid in order to generate the final plasmids pcDNA3.1/EG(DE_4x_-4A/4B)GLuc and pcDNA3.1/EG(KDEL-DE_4x_-4A/4B)GLuc. Huh-7.5 cells were transfected with the jetPEI™ transfection reagent (Polyplus Transfection™, Illkirch, France) according to the manufacturer’s instructions and 24 h post-transfection the cell medium was supplemented with 500 µg/ml G418 (Sigma-Aldrich, St. Louis, MO). At 4 weeks post-selection, bulk Huh-7.5 cell populations were sorted for high EGFP expression using a FACSAria sorter (Becton Dickinson Biosciences, Franklin Lakes, NJ) in order to generate the Huh-7.5/EG(4A/4B)GLuc cell line.

## HCVpp production and infection of Huh-7.5 and Huh-7.5/EG(4A/4B)GLuc cells

HCV pseudoparticles were generated by cotransfection of 293T cells with the jetPEI™ transfection reagent (Polyplus Transfection™, Illkirch, France) with equal amounts of CMV-driven expression cassette plasmids of the viral gps from the Con1 (Acc. No. AJ238799), or HC-J6CF (Acc. No. D00944) or JFH-1 (Acc. No. AB047639) isolates, or Vesicular Stomatitis Virus Glycoprotein (VSV-G ), or an empty vector (no ENV) and the envelope-defective pNL4.3.Luc.R^-^E^-^ proviral HIV genome, according to the manufacturer’s protocols. The supernatants were collected 48 h post-transfection, and were then cleared by passing them through 0.45-μm-pore-size filters. HIV p24 antigen content was assessed by using the commercially available p24 EIA – Innotest™ HIV Antigen mAb (Innogenetics, Gent, Belgium). Normalized supernatants (equal amounts of p24) were used to inoculate Huh-7.5 or Huh-7.5/EG(4A/4B)GLuc cells for 6 h, and were then seeded 18 h prior to inoculation in 12 well plates, 8 x 10^4^ cells/well. Cells were lysed 72 h post-inoculation and luciferase activity was measured as described above.

*Flow cytometry*

Cells were detached by using PBS supplemented with 0.2% EDTA, washed twice with PBS, and passed through a 16-gauge needle. 5 x 10^5^ cells were stained for 1 h at 4^o^C with CD81-specific (clone JS-81) or scavenger receptor class B type I (SR-BI)-specific monoclonal antibodies (clone anti-CLA1) or mouse IgG1, κ isotype control clone MOPC-31C (all antibodies purchased by BD Pharmingen, San Diego, CA) diluted to 10 µg/ml in PBS containing 0.2% bovine serum albumin and 0.02% sodium azide (FACS sample buffer). Subsequently, cells were washed with PBS and bound antibodies were detected by incubation for 1 h at 4^o^C with mouse-specific secondary antibodies conjugated with FITC (Jackson Immunoresearch, Suffolk, UK) at a dilution of 1:100 in FACS sample buffer. Stained cells were washed with PBS, resuspended in 500 μl FACS sample buffer and analyzed using a FACScalibur apparatus and Cell Quest Pro Software (both from Becton Dickinson Biosciences, Franklin Lakes, NJ).

*Transient HCV replication using Firefly luciferase reporter genomes*

Huh-7.5 cells were electroporated with 10 µg RNA of a bicistronic *Firefly* luciferase replicon carrying the JFH-1 genome (kindly provided by Dr. Ralf Bartenschlager) and resuspended in 10 ml DMEM complete. 0.5 ml of the suspension was seeded per well in a 12-well plate for harvesting at 4, 24, 48, 72, and 96 h after transfection (always in duplicate). To assay the luciferase activity, the Luciferase Assay System (Promega Madison, WI) was used according to the manufacturer’s protocol. Measurements were conducted for 10 s using an Orion II Microplate Luminometer (Berthold Detection Systems, Pforzheim, Germany).

*HCV RNA quantification by RT-qPCR*

Viral RNA was isolated from serum- or HCVcc-inoculated cells using the Nucleo Spin RNAII kit (Macherey-Nagel, Düren, Germany) following the manufacturer’s protocol. RNA concentration was determined by measuring the optical density at 260 nm. Twenty-five ng of the total RNA sample was used for quantitative RT-qPCR analysis with an Abbott m2000 Real-Time platform (Abbott Molecular, Wiesbaden, Germany). HCV-specific RT-qPCRs were conducted in triplicate with the OneStep RT-PCR kit (QIAGEN, Hilden, Germany) using the following 5´ NTR-specific probe: S-292, 5´-6-carboxyfluorescein- CCTGATAGGGTGCTTGCGAGTGCC -tetrachloro-6-carboxyfluorescein-3´; and primers; S-271, 5´- GCGAAAGGCCTTGTGGTACT-3´; and A-337, 5´- CACGGTCTACGAGACCTCCC -3´ (Biomers, Ulm, Germany). Reactions were performed in three stages under the following conditions: stage 1, 60 min at 55°C (reverse transcription); stage 2, 15 min at 95°C (heat inactivation of reverse transcriptase and activation of Taq polymerase); and stage 3, 40 cycles of 15 s at 95°C and 1 min 60°C (amplification). The total volume of the reaction mix was 15 µl and it contained the following components: 2.66 μM 6-carboxy-X-rhodamine (passive reference), 4 mM MgCl_2_, 0.66 mM deoxynucleoside triphosphates, 0.266 µM HCV probe, 1 µM of each HCV primer, and 0.6 µl enzyme mix. The amount of HCV RNA was calculated by comparing it to serially diluted *in vitro* transcripts.

*Preparation of cell lysates, PAGE, and Western blot analysis*

In order to prepare cell lysates for Western blot analysis, naïve or infected cells were lysed with 1% Triton X-100 in PBS and clarified by centrifugation at 13,000 rpm for 10 min. Debris-free supernatants were quantified with a Lowry protein assay, and an equal amount of protein for each sample was diluted into sample buffer (160 mM Tris, pH 6.7, 2% SDS, 700 mM β-mercaptoethanol, 10% glycerol, 0.004% bromophenol blue). Samples were heated at 70°C for 10 min and then loaded onto a 4-12% SDS-polyacrylamide gel (NuPAGE®, Novex 4-12%, Bis-Tris Midi gels, Invitrogen, Eugene, OR). Following electrophoresis, proteins were transferred to a polyvinylidine fluoride membrane (PerkinElmer, Life Sciences, CA). Blots were blocked overnight at 4°C in blocking solution (5% milk powder and 0.05% Tween20 in PBS). Incubation with the primary receptor-specific antibodies (mouse anti-occludin or rabbit anti-claudin-1, both at a final concentration of 1 μg/ml; Invitrogen, Eugene, OR), or HCV protein-specific antibodies (core protein with anti-core antibody C7-50, Santa Cruz Biotechnologies, Santa Cruz, CA at 0.2 μg/ml and NS3 protein with anti-NS3 antibody clone H23, Abcam, Cambridge, UK at .1 μg/ml), or β-actin (anti-β-actin mouse mAb AM4302 Ambion, Austin, TX, at 0.01 μg/ml) or GAPDH-specific at 0.025 μg/ml (anti-GAPDH mouse mAb, clone 6C5; Applied Biosystems, Foster city, CA), or EGFP-specific (anti-GFP clone GSN149, Sigma-Aldrich,St. Louis, MO, at 1 μg/ml) was performed in blocking solution for 1 h at room temperature. Blots were washed five times for 10 min in washing solution (0.05% Tween20 in PBS), incubated for 1 h with anti-mouse (1:5,000, Sigma-Aldrich, St. Louis, MO) or anti-rabbit horseradish peroxidase-conjugated secondary antibodies (1:5,000; Jackson Immunoresearch, Suffolk, UK) in blocking solution, and washed as described above. Antibody-protein complexes were detected using the SuperSignal® West Femto Maximum Sensitivity Substrate Kit (Thermo Fischer Scientific Inc., MA)

# References

1. Kalajzic I, Stover ML, Liu P, Kalajzic Z, Rowe DW, et al. (2001) Use of VSV-G pseudotyped retroviral vectors to target murine osteoprogenitor cells. Virology 284: 37-45.
